# Supplementary material for: Characterization, Classification, and Authentication of Polygonatum sibiricum Samples by Volatile Profiles and Flavor Properties
Source: Molecules. 2021 Dec 21;27(1):25. doi: 10.3390/molecules27010025 (PMC8746527; doi:10.3390/molecules27010025)
Supplement: Supplementary file 1 [file molecules-27-00025-s001.zip › molecules-1485341-supplementary.pdf]

Supplementary material

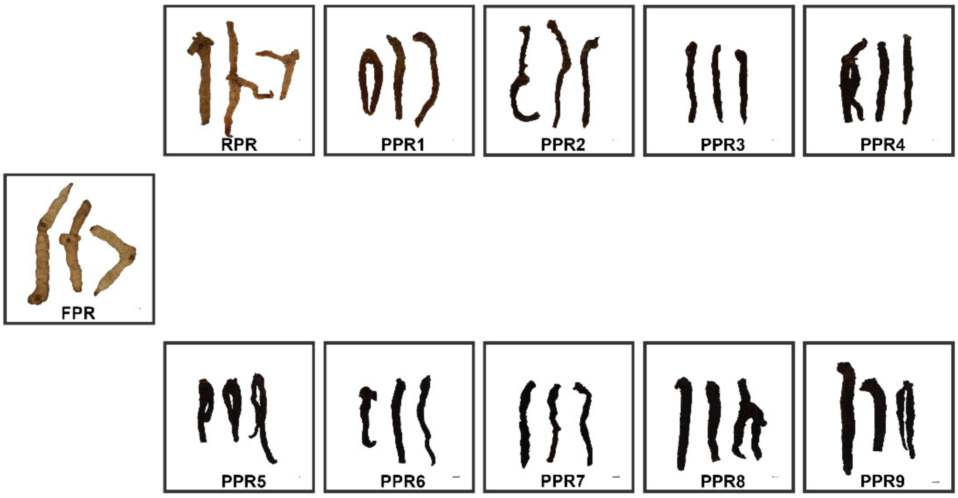

Figure S1. Appearance characteristics of FPR and PR samples with different processing levels.

Table S1. Sensors used in PEN3 electronic nose and their performance description.

Array number	Sensors	Substances for sensing
1	W1C	Sensitive to aromatic compounds
2	W5S	Sensitive to nitrogen oxides
3	W3C	Sensitive to ammonia and aromatic compounds
4	W6S	Sensitive to hydrogen
5	W5C	Sensitive to hydrocarbons, aromatic compounds
6	W1S	Sensitive to methane in the environment, with broad range
7	W1W	Sensitive to sulfur compounds, pyrazine, many terpenes, such as limonene
8	W2S	Sensitive to ethanol, some aromatic compounds, broad range
9	W2W	Sensitive to aromatic components, sulfur compounds
10	W3S	Sensitive to methane and some high concentration compounds

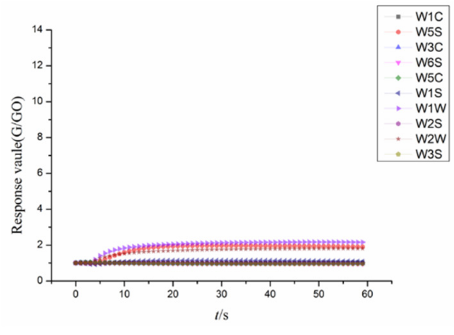

RPR

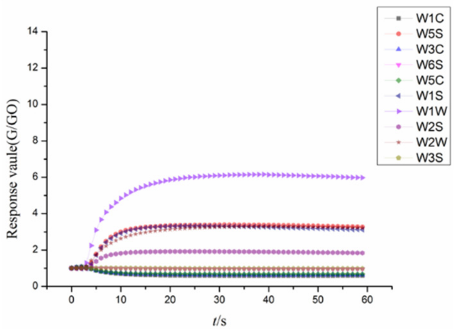

PPR1

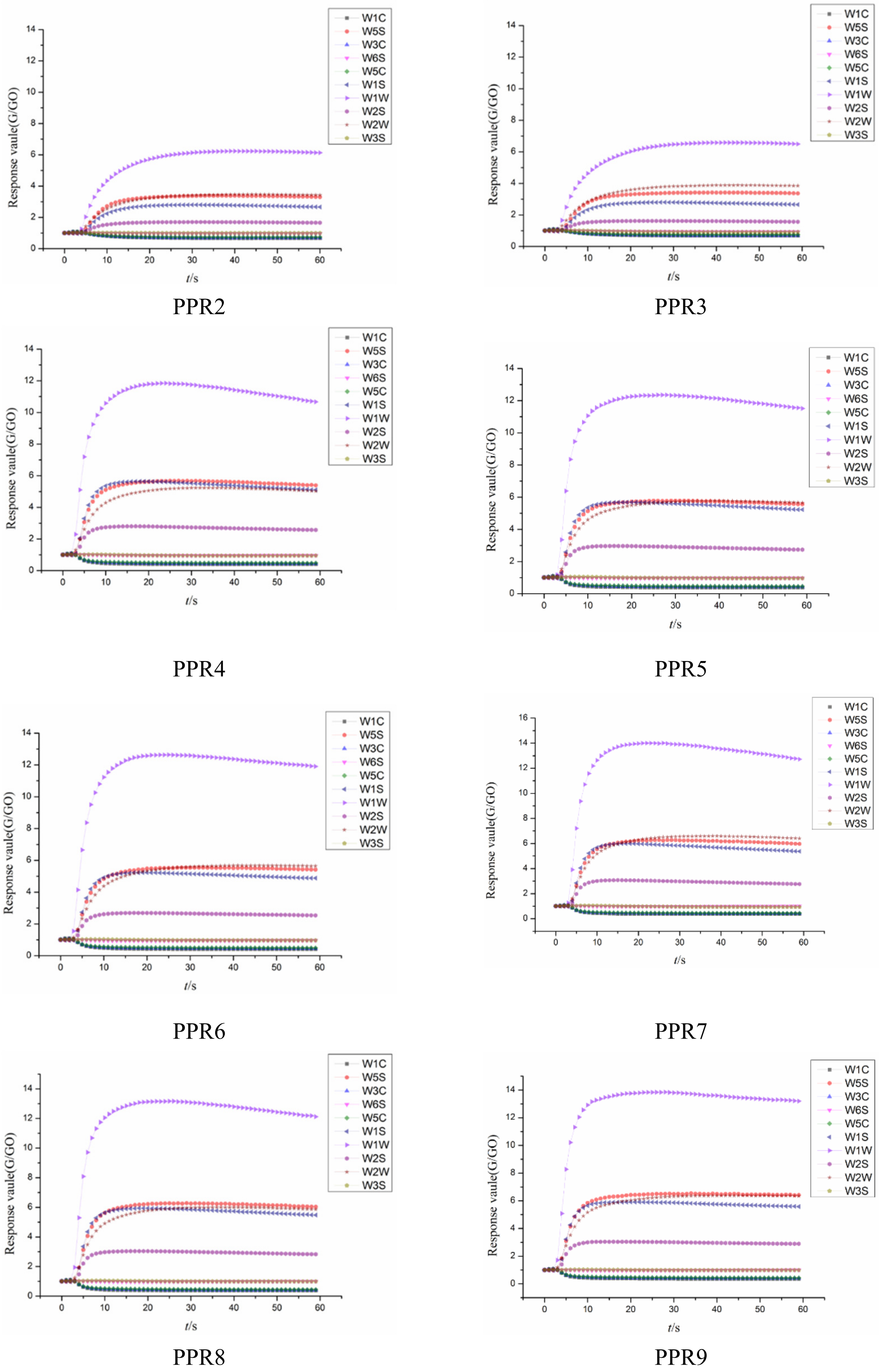

Figure S2. Odor response curves of PR samples with different processing levels.

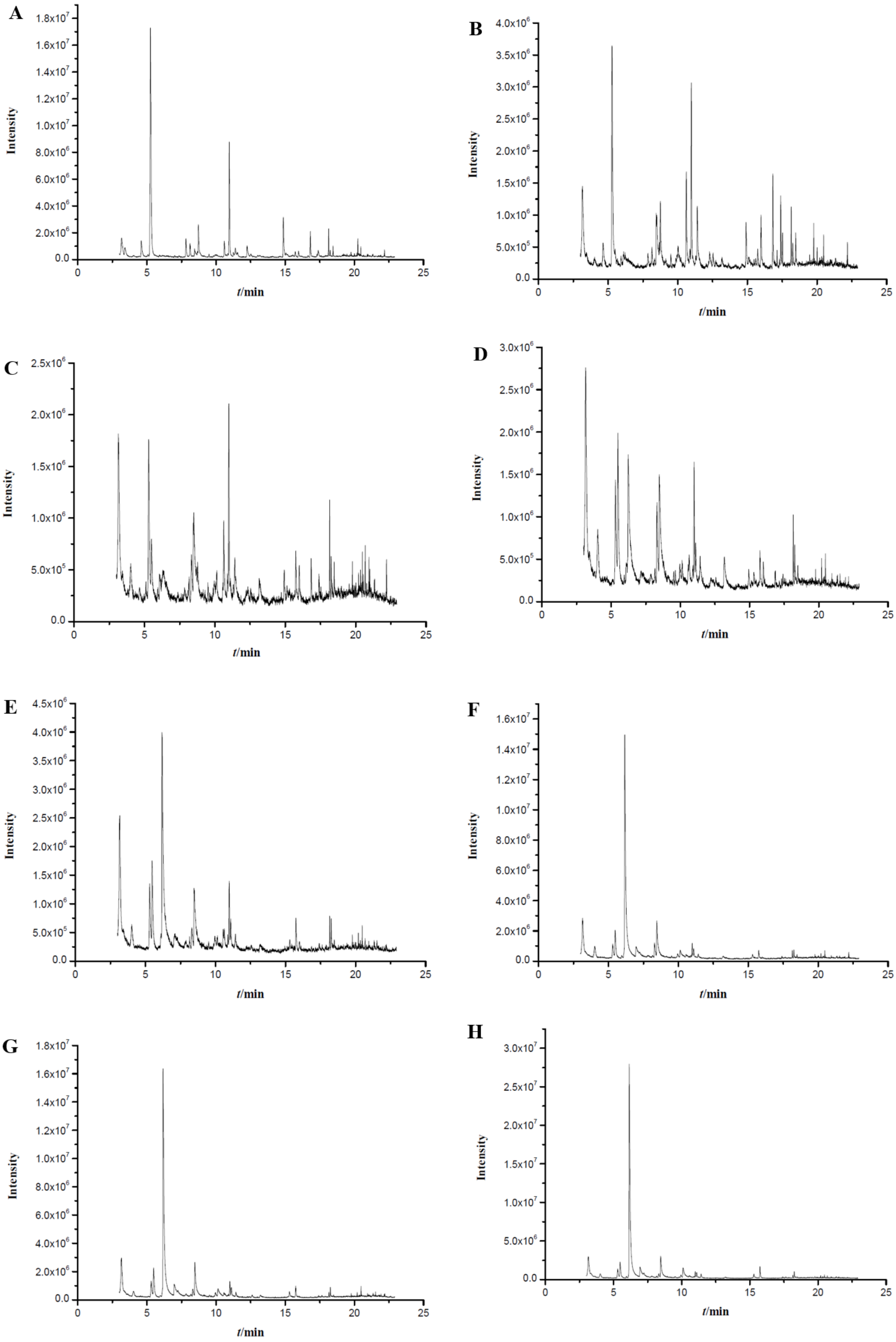
A
B
C
D
E
F
G
H
Intensity
t/min
0
5
10
15
20
25

I

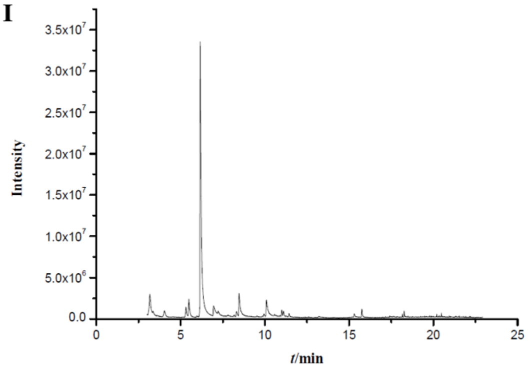

J

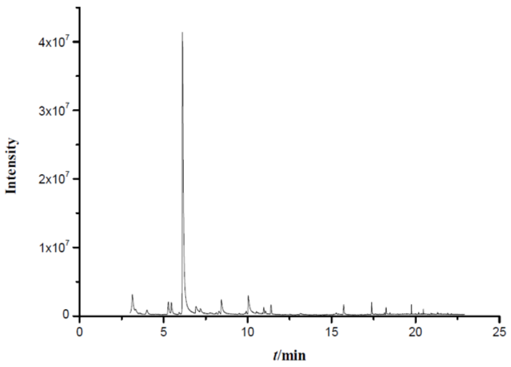

Figure S3. GC–MS profile of PR samples with different processing levels. (A) RPR. (B) PPR1. (C) PPR2. (D) PPR3. (E) PPR4. (F) PPR5. (G) PPR6. (H) PPR7. (I) PPR8. (J) PPR9.

Table S2. Odor response values of PR samples with different processing levels.

Sensors	Response values: Average (minimum–maximum)											Significance
	RPR	PPR1	PPR2	PPR3	PPR4	PPR5	PPR6	PPR7	PPR8	PPR9	*p* value	
W1C	0.976 (0.960–0.988)	1.656 (1.614–1.697)	1.472 (1.439–1.505)	1.530 (1.466–1.582)	2.408 (2.387–2.431)	2.585 (2.564–2.603)	2.462 (2.434–2.514)	2.665 (2.644–2.680)	2.698 (2.690–2.706)	2.737 (2.699–2.765)	0.000	***
W5S	1.948 (1.923–1.975)	3.209 (3.125–3.292)	3.260 (3.192–3.322)	3.516 (3.383–3.620)	4.957 (4.847–5.130)	5.578 (5.561–5.606)	5.477 (5.419–5.558)	6.034 (6.015–6.048)	6.094 (6.050–6.168)	6.286 (6.092–6.424)	0.000	***
W3C	1.017 (1.006–1.024)	1.552 (1.518–1.586)	1.429 (1.404–1.454)	1.499 (1.445–1.542)	2.169 (2.143–2.211)	2.382 (2.354–2.396)	2.275 (2.251–2.320)	2.473 (2.444–2.489)	2.489 (2.460–2.514)	2.506 (2.465–2.535)	0.000	***
W6S	0.958 (0958–0.959)	0.948 (0.947–0.950)	0.956 (0.953–0.960)	0.939 (0.938–0.939)	0.964 (0.962–0.965)	0.972 (0.971–0.973)	0.963 (0.960–0.968)	0.996 (0.996–0.997)	1.000 (0.997–1.003)	0.999 (0.994–1.001)	0.001	**
W5C	0.998 (0.992–1.002)	1.446 (1.418–1.473)	1.334 (1.312–1.357)	1.388 (1.343–1.423)	1.984 (1.959–2.028)	2.208 (2.155–2.237)	2.064 (2.038–2.110)	2.303 (2.254–2.328)	2.297 (2.254–2.329)	2.306 (2.279–2.325)	0.000	***
W1S	1.078 (1.042–1.101)	3.051 (2.960–3.143)	2.614 (2.534–2.686)	2.814 (2.673–2.923)	4.923 (4.850–5.000)	5.256 (5.222–5.298)	4.961 (4.878–5.092)	5.394 (5.300–5.461)	5.479 (5.451–5.496)	5.583 (5.551–5.611)	0.000	***
W1W	2.207 (2.165–2.253)	5.738 (5.470–6.005)	6.002 (5.827–6.164)	6.880 (6.523–7.159)	9.792 (9.528–10.141)	11.603 (11.550 – 11.696)	12.008 (11.925–12.078)	12.956 (12.884–13.002)	13.190 (13.016–13.324)	12.873 (12.337–13.263)	0.000	***
W2S	0.979 (0.965–0.992)	1.807 (1.765–1.849)	1.622 (1.576–1.664)	1.626 (1.568–1.671)	2.423 (2.381–2.482)	2.729 (2.689–2.763)	2.576 (2.541–2.629)	2.770 (2.725–2.801)	2.804 (2.772–2.825)	2.879 (2.856–2.897)	0.000	***
W2W	1.782 (1.760–1.801)	3.147 (3.051–3.243)	3.369 (3.2943.442).	4.017 (3.855–4.142)	4.564 (4.444–4.754)	5.568 (5.347–5.691)	5.655 (5.641–5.667)	6.470 (6.441–6.498)	6.512 (6.364–6.680)	6.168 (5.899–6.358)	0.000	***
W3S	0.948 (0.940–0.953)	0.978 (0.978–0.979)	0.995 (0.995–0.996)	0.897 (0.888–0.910)	0.938 (0.935–0.939)	0.959 (0.955–0.966)	0.968 (0.966–0.971)	0.938 (0.935–0.943)	0.960 (0.952–0.969)	0.989 (0.988–0.989)	0.001	**

Table S3. Taste response values of PR samples with different processing levels.

Taste	Response values: Average (minimum, maximum)											Significance
	RPR	PPR1	PPR2	PPR3	PPR4	PPR5	PPR6	PPR7	PPR8	PPR9	*p* value	
Sourness	–40.94<br>(-41.64, -40.56)	-34.83<br>(-35.09, -34.33)	-31.93<br>(-31.79, -30.71)	-27.52<br>(-28.01, -26.78)	-26.98<br>(-27.56, -6.22)	-24.58<br>(-25.19, -23.76)	-20.02<br>(-20.76, -19.22)	-14.80<br>(-15.55, -13.98)	-14.98<br>(-15.76, -14.11)	-10.95<br>(-11.76, -10.16)	0.000	***
Bitterness	8.58<br>(8.55, 8.62)	7.05<br>(7.00, 7.15)	6.85<br>(6.75, 7.00)	6.63<br>(6.49, 6.79)	6.55<br>(6.43, 6.69)	8.17<br>(8.15, 8.22)	7.87<br>(7.75, 8.04)	8.09<br>(7.75, 8.67)	7.03<br>(6.96, 7.14)	6.02<br>(5.85, 6.19)	0.001	**
Astringency	-0.40<br>(-0.43, -0.35)	-0.21<br>(-0.23, -0.20)	0.71<br>(0.56, 0.88)	0.21<br>(0.17, 0.29)	0.29<br>(0.22, 0.42)	0.59<br>(0.57, 0.62)	0.73<br>(0.66, 0.82)	1.29<br>(1.20, 1.37)	1.02<br>(0.97, 1.06)	2.17<br>(1.98, 2.35)	0.000	***
Aftertaste-B	-0.22<br>(-0.26, -0.19)	-0.23<br>(-0.29, -0.18)	0.33<br>(0.28, 0.41)	-0.08<br>(-0.14, -0.05)	0.04<br>(0.02, 0.05)	0.01<br>(0.01, 0.02)	0.05<br>(0.02, 0.08)	0.09<br>(0.08, 0.10)	0.08<br>(0.06, 0.09)	0.48<br>(0.47, 0.49)	0.001	**
Aftertaste-A	0.49<br>(0.46, 0.54)	0.65<br>(0.62, 0.67)	0.80<br>(0.78, 0.81)	0.69<br>(0.67, 0.71)	0.68<br>(0.66, 0.70)	0.62<br>(0.61, 0.65)	0.69<br>(0.65, 0.73)	0.73<br>(0.68, 0.76)	0.62<br>(0.60, 0.63)	0.86<br>(0.85, 0.87)	0.000	***
Umami	7.78<br>(7.75, 7.80)	5.63<br>(5.27, 5.83)	5.07<br>(4.68, 5.35)	3.47<br>(3.11, 3.70)	3.52<br>(3.13, 3.81)	1.53<br>(1.16, 1.81)	1.58<br>(1.11, 1.98)	0.44<br>(0.35, 0.58)	-0.63<br>(-0.74, -0.52)	-0.54<br>(-0.63, -0.48)	0.000	***
Richness	-0.75<br>(-0.79, -0.73)	-0.74<br>(-0.79, -0.66)	-0.66<br>(-0.67, -0.64)	-0.66<br>(-0.69, -0.63)	-0.66<br>(-0.69, -0.64)	-0.55<br>(-0.57, -0.53)	-0.56<br>(-0.58, -0.54)	-0.38<br>(-0.41, -0.35)	-0.52<br>(-0.55, -0.50)	-0.38<br>(-0.41, -0.35)	0.000	***
Saltiness	-4.23<br>(-4.26, -4.20)	-3.97<br>(-4.09, -3.90)	-1.11<br>(-1.31, -1.00)	-5.60<br>(-5.69, -5.53)	-4.87<br>(-5.05, -4.76)	-7.44<br>(-7.50, -7.39)	-6.86<br>(-6.97, -6.75)	-8.53<br>(-8.63, -8.43)	-7.33<br>(-7.92, -7.00)	-6.16<br>(-6.30, -5.99)	0.001	**
Sweetness	15.15<br>(15.12, 15.18)	13.79<br>(13.76, 13.83)	12.09<br>(12.03, 12.16).	11.37<br>(11.34, 11.40)	11.23<br>(11.20,11.28)	11.11<br>(11.09,11.13)	9.28<br>(9.23, 9.30)	9.40<br>(9.39, 9.42)	9.72<br>(9.67, 9.76)	8.26<br>(8.22, 8.30)	0.000	***

Table S4. Number of volatile compounds in PR samples with different processing levels.

Name	RPR	PPR1	PPR2	PPR3	PPR4	PPR5	PPR6	PPR7	PPR8	PPR9
Amines	1	1	1	1	1	0	0	0	0	0
Alcohols	4	3	3	3	3	3	3	3	3	3
Phenols	2	2	2	2	2	2	2	2	2	2
Furans/Pyrans	6	6	8	8	8	10	10	10	10	10
Nitrogen heterocycles	3	3	4	4	3	3	3	3	3	3
Sulfides	1	2	2	2	2	2	2	2	2	2
Aldehydes	6	6	5	6	6	5	5	5	5	5
Carboxylic acids	1	1	1	1	1	1	1	1	1	1
Terpenoids	6	9	9	7	6	5	5	4	4	4
Ketones	3	3	3	4	4	3	3	3	3	3
Alkanes/Alkenes	5	4	4	4	4	4	4	4	4	4
Esters	1	1	1	1	1	1	1	1	1	1

Table S5. Relative content of volatile compounds in PR samples with different processing levels.

Name	RPR	PPR1	PPR2	PPR3	PPR4	PPR5	PPR6	PPR7	PPR8	PPR9
Amine	2.11%	4.57%	1.88%	0.61%	0.20%	0%	0%	0%	0%	0%
Alcohol	3.92%	11.90%	9.22%	3.13%	2.29%	0.63%	0.76%	0.52%	0.39%	0.48%
Phenol	0.38%	0.66%	0.55%	0.64%	0.70%	0.79%	0.97%	0.69%	0.64%	0.58%
Furan/Pyran	22.60%	21.92%	21.74%	28.95%	39.22%	67.39%	66.30%	74.55%	77.32%	78.76%
Nitrogen heterocycle	3.55%	3.53%	5.48%	11.45%	21.64%	8.30%	8.82%	7.33%	7.19%	5.90%
Sulfide	0.06%	0.64%	2.59%	3.08%	1.63%	2.01%	0.90%	0.74%	0.99%	0.66%
Aldehyde	35.56%	23.72%	16.90%	9.96%	7.17%	3.85%	4.51%	3.70%	2.91%	3.30%
Carboxylic acid	0.61%	1.48%	2.15%	1.11%	1.23%	0.57%	1.13%	0.36%	0.28%	0.28%
Terpenoid	2.53%	3.55%	3.74%	1.81%	1.02%	0.40%	0.42%	0.28%	0.26%	0.25%
Ketone	6.45%	13.01%	25.11%	26.78%	20.29%	13.81%	13.79%	10.12%	8.41%	7.81%
Alkane/Alkene	22.16%	14.36%	10.24%	5.95%	4.36%	2.17%	2.52%	1.64%	1.55%	1.73%
Ester	0.09%	0.16%	0.22%	0.17%	0.12%	0.08%	0.08%	0.06%	0.05%	0.06%

Table S6. The relative content of common compounds in PR samples with different processing levels.

NO.	Retention Time(min)	Compounds	Relative content（%）									
			RPR	PPR1	PPR2	PPR3	PPR4	PPR5	PPR6	PPR7	PPR8	PPR9
1	3.160	Pinacolone	2.713±0.034	10.390±0.105	22.947±1.197	25.619±2.460	19.570±0.933	13.186±0.253	13.067±0.509	9.342±0.141	7.495±0.494	6.659±0.084
2	3.245	3-Methylcyclohexene	0.430±0.012	0.610±0.032	0.646±0.015	0.478±0.005	0.319±0.022	0.161±0.002	0.153±0.009	0.096±0.007	0.183±0.019	0.086±0.005
3	4.028	Dimethyl disulfide	0.060±0.004	0.395±0.045	1.684±0.235	2.429±0.336	1.257±0.105	1.727±0.016	0.768±0.067	0.654±0.023	0.909±0.044	0.592±0.086
4	4.643	N-Nitrosopiperidine	2.912±0.031	1.844±0.030	0.941±0.005	0.381±0.024	0.251±0.007	0.108±0.002	0.122±0.007	0.083±0.004	0.066±0.005	0.072±0.009
5	5.109	3,4-Dihydro-2H-pyran	0.055±0.012	0.162±0.011	1.235±0.038	0.371±0.057	0.169±0.006	0.099±0.009	0.116±0.006	0.069±0.007	0.056±0.005	0.052±0.004
6	5.299	Hexanal	29.883±0.287	18.477±0.513	11.549±0.552	6.211±0.295	4.571±0.186	2.204±0.036	2.403±0.102	1.710±0.036	1.508±0.089	1.895±0.088
7	5.302	Octane	21.094±0.101	13.049±0.340	8.599±0.542	4.789±0.293	3.521±0.248	1.666±0.064	1.733±0.077	1.291±0.043	1.174±0.044	1.400±0.030
8	6.092	2-Aminopyridine	0.111±0.014	1.149±0.040	2.341±0.244	1.449±0.226	0.808±0.047	0.363±0.011	0.313±0.021	0.146±0.008	0.088±0.001	0.060±0.005
9	7.830	1-Methylcyclohexene	0.365±0.003	0.215±0.045	0.194±0.013	0.182±0.008	0.153±0.007	0.096±0.006	0.101±0.007	0.059±0.002	0.058±0.002	0.056±0.004
10	7.860	2-Butylfuran	0.599±0.011	0.451±0.032	0.445±0.032	0.356±0.023	0.244±0.013	0.091±0.012	0.101±0.010	0.075±0.003	0.052±0.008	0.069±0.006
11	7.873	2-Heptanone	3.376±0.099	1.558±0.047	0.935±0.016	0.397±0.035	0.254±0.022	0.067±0.005	0.076±0.008	0.042±0.000	0.033±0.003	0.025±0.001
12	9.514	Camphene	0.176±0.017	0.555±0.006	0.584±0.053	0.348±0.016	0.200±0.013	0.122±0.011	0.117±0.014	0.074±0.006	0.064±0.010	0.055±0.005
13	9.955	Benzaldehyde	0.213±0.013	0.662±0.032	1.016±0.024	0.795±0.128	0.578±0.029	0.432±0.014	0.491±0.007	0.333±0.013	0.309±0.017	0.336±0.008
14	10.631	1-Octen-3-OL	3.485±0.185	11.466±0.645	8.659±0.519	2.631±0.143	1.836±0.134	0.393±0.019	0.444±0.020	0.230±0.015	0.182±0.014	0.255±0.013
15	10.986	2-Pentylfuran	21.068±0.580	19.848±0.304	16.456±0.676	9.151±0.219	6.831±0.308	2.813±0.115	3.133±0.141	1.754±0.067	1.571±0.110	1.772±0.083
16	11.098	Benzofuran	0.046±0.008	0.377±0.024	1.098±0.050	2.125±0.174	1.699±0.050	1.328±0.056	1.403±0.053	0.975±0.019	0.803±0.059	0.470±0.029
17	12.593	2-Ethylhexanol	0.191±0.020	0.270±0.013	0.202±0.027	0.193±0.022	0.167±0.011	0.085±0.008	0.087±0.002	0.065±0.005	0.053±0.003	0.052±0.003
18	15.430	Methyl benzoate	0.086±0.018	0.162±0.021	0.218±0.024	0.172±0.016	0.120±0.012	0.085±0.008	0.083±0.005	0.056±0.003	0.049±0.001	0.058±0.005
19	15.485	Isopulegol	0.058±0.009	0.155±0.020	0.219±0.034	0.152±0.009	0.123±0.012	0.080±0.013	0.085±0.004	0.042±0.010	0.049±0.007	0.058±0.005
20	15.746	Nonanal	0.436±0.011	0.853±0.028	2.001±0.072	1.457±0.091	1.340±0.138	0.777±0.052	1.127±0.156	1.272±0.102	0.794±0.044	0.872±0.041
21	15.746	2-Methylcyclohexanol	0.071±0.009	0.166±0.017	0.361±0.030	0.302±0.010	0.285±0.029	0.149±0.017	0.229±0.030	0.223±0.003	0.151±0.007	0.176±0.012
22	17.621	Menthol	0.144±0.026	0.241±0.011	0.231±0.010	0.307±0.014	0.307±0.024	0.097±0.009	0.111±0.015	0.128±0.016	0.114±0.008	0.096±0.031
23	18.487	Beta-Cyclocitral	0.110±0.008	0.356±0.046	0.308±0.035	0.312±0.032	0.184±0.005	0.168±0.003	0.134±0.014	0.068±0.007	0.061±0.004	0.097±0.006
24	19.768	Aminocaproic acid	0.607±0.047	1.479±0.194	2.153±0.724	1.109±0.231	1.134±0.049	0.567±0.092	±0.046	0.363±0.025	0.277±0.050	0.275±0.057

25	20.259	Longifolene	0.944±0.102	0.145±0.020	0.165±0.008	0.124±0.032	0.113±0.014	0.054±0.002	0.060±0.005	0.040±0.004	0.037±0.003	0.037±0.005
26	21.348	Pentadecane	0.215±0.015	0.486±0.038	0.806±0.093	0.497±0.047	0.362±0.026	0.251±0.032	0.537±0.083	0.196±0.013	0.135±0.011	0.186±0.006

Table S7. The relative content of new compounds in different processing levels.

NO.	Retention Time(min)	Compounds	Relative content（%）									
			RPR	PPR1	PPR2	PPR3	PPR4	PPR5	PPR6	PPR7	PPR8	PPR9
1	3.354	2,5-Dimethylfuran	ND	ND	0.143±0.013	0.158±0.012	0.176±0.007	0.280±0.018	0.314±0.011	0.396±0.012	0.444±0.028	0.439±0.024
2	6.126	M-Cresol	ND	ND	ND	0.349±0.015	0.486±0.021	0.647±0.031	0.683±0.034	0.600±0.014	0.543±0.047	0.500±0.033
3	6.176	Furfural	ND	ND	ND	9.783±1.011	20.916±0.805	49.655±1.241	49.114±0.940	60.093±0.240	62.852±0.782	67.191±1.176
4	6.178	2,5-Furandione	ND	0.606±0.035	0.969±0.113	1.429±0.142	1.284±0.048	1.058±0.025	0.925±0.013	0.839±0.034	0.734±0.036	0.821±0.023
5	6.271	1,4-Dimethylpyrazole	ND	ND	1.263±0.119	9.203±1.028	20.577±0.261	7.832±0.408	8.383±0.654	7.104±0.357	7.040±0.289	5.766±1.584
6	6.960	2-Furanmethanol	ND	ND	ND	ND	ND	0.855±0.078	0.983±0.005	1.100±0.046	0.938±0.082	0.709±0.077
7	7.223	Cyclohexanone	ND	ND	ND	0.112±0.017	0.319±0.011	0.553±0.087	0.646±0.040	0.739±0.035	0.887±0.009	1.124±0.012
8	8.473	2-Ethyl-5-methylfuran	ND	ND	1.057±0.034	5.578±0.432	7.899±0.254	10.639±0.265	9.547±0.647	7.722±0.158	7.374±0.409	4.626±0.179
9	10.103	5-Methylfurfural	ND	ND	ND	ND	ND	0.577±0.020	0.662±0.063	1.531±0.066	2.496±0.161	2.608±0.096
10	10.132	Dimethyl trisulfide	ND	0.247±0.035	0.903±0.251	0.653±0.085	0.373±0.061	0.281±0.018	0.129±0.015	0.086±0.003	0.083±0.012	0.064±0.002
11	15.302	2-Ethyl-2-hexenal	ND	ND	ND	0.281±0.065	0.267±0.027	0.265±0.032	0.359±0.013	0.317±0.020	0.240±0.010	0.103±0.011
12	17.547	Geraniol	ND	0.942±0.043	0.211±0.034	0.161±0.016	ND	ND	ND	ND	ND	ND
13	20.030	(+)-Alpha-Muurolene	ND	0.265±0.005	0.161±0.008	ND	ND	ND	ND	ND	ND	ND
14	20.787	Beta-Caryophyllene	ND	0.109±0.007	0.296±0.040	ND	ND	ND	ND	ND	ND	ND
15	21.523	Butylated hydroxytoluene	ND	ND	ND	0.294±0.027	0.217±0.021	0.147±0.018	0.288±0.040	0.089±0.009	0.097±0.015	0.083±0.008

ND, non-detectable.

Table S8. The relative content of disappeared compounds in different processing levels.

No.	Retention Time(min)	Compounds	Relative content（%）									
			RPR	PPR1	PPR2	PPR3	PPR4	PPR5	PPR6	PPR7	PPR8	PPR9
1	7.329	3-Methyl-1-pentanol	0.169±0.003	ND	ND	ND	ND	ND	ND	ND	ND	ND
2	8.698	Alpha-Pinene	0.521±0.033	1.022±0.042	0.963±0.046	0.353±0.008	0.142±0.018	0.047±0.003	0.049±0.002	ND	ND	ND
3	9.148	2,6-Dimethylphenol	0.066±0.004	0.223±0.007	ND	ND	ND	ND	ND	ND	ND	ND
4	11.259	Undecane	0.056±0.007	ND	ND	ND	ND	ND	ND	ND	ND	ND
5	11.296	4-Dimethylaminopyridine	0.527±0.020	0.540±0.148	0.623±0.121	0.413±0.099	ND	ND	ND	ND	ND	ND
6	11.377	4-Methoxyphenylacetone	0.360±0.042	1.065±0.057	1.231±0.145	0.648±0.082	0.151±0.005	ND	ND	ND	ND	ND
7	14.922	3-Methoxybenzaldehyde	4.545±0.553	3.130±0.104	2.027±0.261	0.908±0.137	0.234±0.008	ND	ND	ND	ND	ND
8	15.067	Allyl 2-furoate	0.644±0.022	0.474±0.007	0.337±0.035	ND	ND	ND	ND	ND	ND	ND
9	16.840	2,4-Dimethylaniline	2.108±0.107	4.572±0.532	1.883±0.142	0.605±0.158	0.205±0.006	ND	ND	ND	ND	ND
10	17.363	Piperonal	0.372±0.033	0.243±0.014	ND	ND	ND	ND	ND	ND	ND	ND
11	17.916	Gamma-Undecalactone	0.184±0.013	ND	ND	ND	ND	ND	ND	ND	ND	ND
12	18.464	Verbenone	0.690±0.047	1.134±0.073	0.914±0.159	0.366±0.029	0.135±0.010	ND	ND	ND	ND	ND
13	19.672	2,5-Dimethylphenol	0.309±0.025	0.439±0.058	0.336±0.064	ND	ND	ND	ND	ND	ND	ND

ND, non-detectable.

Table S9. List of compounds identified in PR samples with different processing levels.

No.	Compound	RI (Ref)	RI (Exp)	Dot product	Reverse dot product
1	Pinacolone	612	601	99.2	73.2
2	3-Methylcyclohexene	643	605	86.1	83
3	2,5-Dimethylfuran	632	610	88.7	75.6
4	Dimethyl disulfide	676	643	96.2	97.4
5	N-Nitrosopiperidine		673	82.8	64.9
6	3,4-Dihydro-2H-pyran	691	695	74	81.1
7	Hexanal	806	801	80.4	81.1
8	Octane	816	802	96.3	72.1
9	2-Aminopyridine	846	815	90.8	82.8
10	M-Cresol	814	815	84.2	86.8
11	Furfural	831	816	85.7	86.7
12	2,5-Furandione	839	816	82.6	71.7
13	1,4-Dimethylpyrazole	804	818	85.8	74.4
14	2-Furanmethanol	885	829	98.4	73
15	Cyclohexanone	891	833	72.9	81
16	3-Methyl-1-pentanol	796	835	89.6	66.6
17	1-Methylcyclohexene	791	843	62.3	92.8
18	2-Butylfuran	841	844	71.6	86.4
19	2-Heptanone	853	844	88	81.6
20	2-Ethyl-5-methylfuran	831	854	91.4	80.7
21	Alpha-Pinene	868	858	96.8	92.6
22	2,6-Dimethylphenol	887	865	66.7	90.9
23	Camphene	904	871	96.7	89.3
24	Benzaldehyde	882	878	91.2	98.1
25	5-Methylfurfural	920	881	99.1	90
26	Dimethyl trisulfide	902	881	81	93.1
27	1-Octen-3-OL	928	889	90.1	82.5
28	2-Pentylfuran	904	895	86.8	90.5
29	Benzofuran	918	897	97.9	93.9
30	Undecane	948	900	97.2	71.6
31	4-Dimethylaminopyridine	1006	1000	84.9	85.2
32	4-Methoxyphenylacetone	1018	1002	68.4	83.4
33	2-Ethylhexanol	1020	1019	97.2	72.4
34	3-Methoxybenzaldehyde	1051	1053	72.4	92.7
35	Allyl 2-furoate	1058	1055	85.1	75.1
36	2-Ethyl-2-hexenal	1045	1059	81.4	63
37	Methyl benzoate	1060	1060	73.8	90.1
38	Isopulegol	1066	1061	70	74.8
39	Nonanal	1089	1065	98.1	71.4
40	2-Methylcyclohexanol	1040	1065	79.6	73.4
41	2,4-Dimethylaniline	1099	1081	94.1	88
42	Piperonal	1106	1089	66.3	80.5
43	Geraniol	1098	1091	74.2	85
44	Menthol	1194	1092	88.9	90.1
45	Gamma-Undecalactone		1097	85.1	61.2
46	Verbenone	1233	1214	89.4	79.7
47	Beta-Cyclocitral	1233	1215	67.3	84.8
48	2,5-Dimethylphenol	1267	1266	68.6	77.2
49	Aminocaproic acid	1267	1270	76.6	92.8
50	(+)-Alpha-Muurolene	1295	1281	80.1	72.1
51	Longifolene	1298	1291	78.3	85.8
52	Beta-Caryophyllene	1417	1419	92.8	88.9
53	Pentadecane	1462	1452	89.6	87.4
54	Butylated hydroxytoluene	1483	1463	78.6	92

1

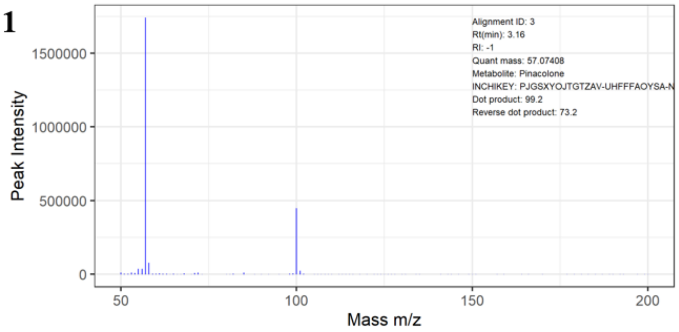

2

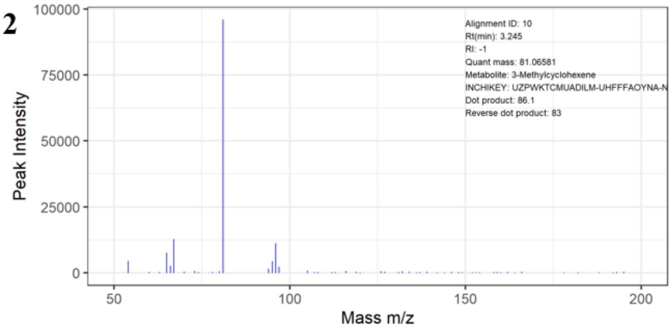

3

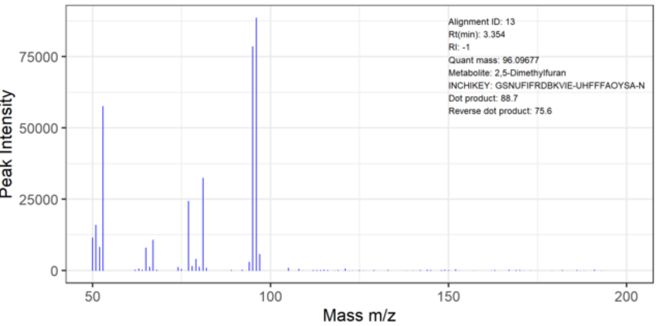

4

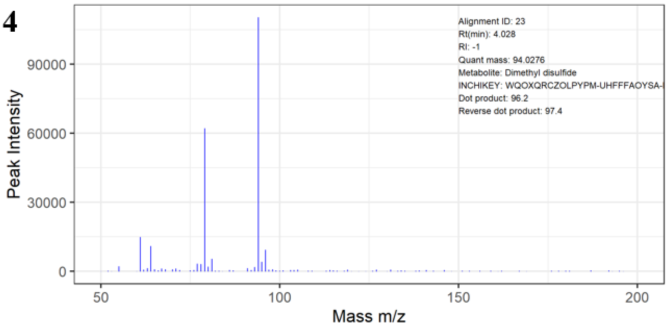

5

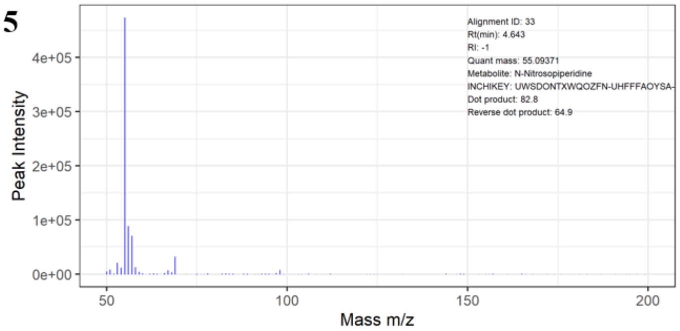

6

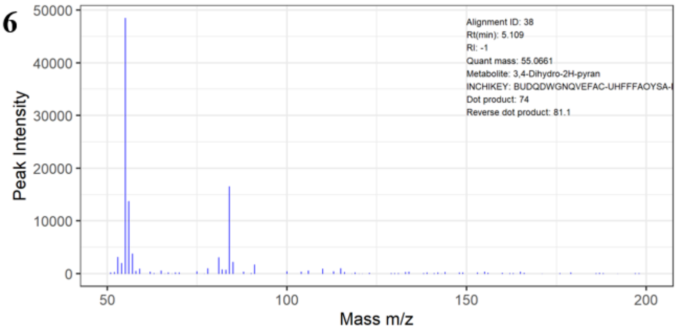

7

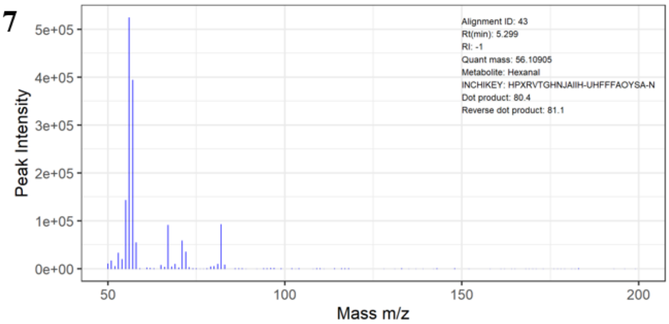

8

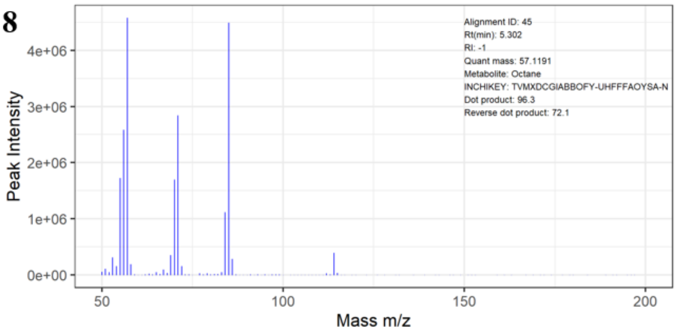

9

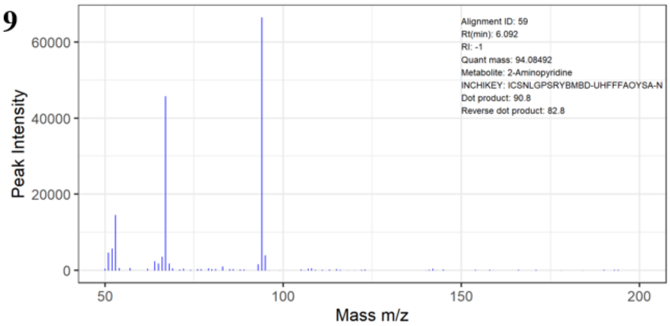

10

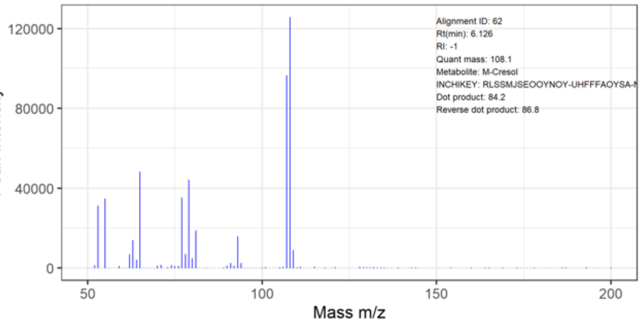

11

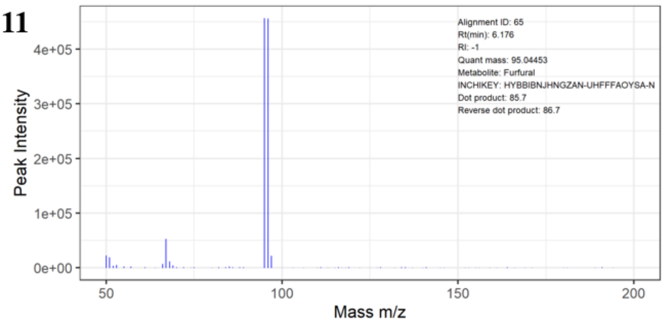

12

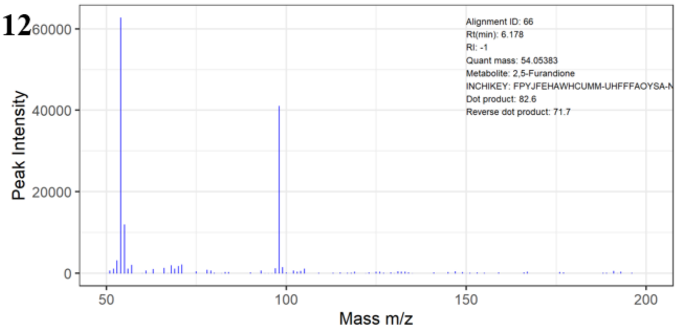

13

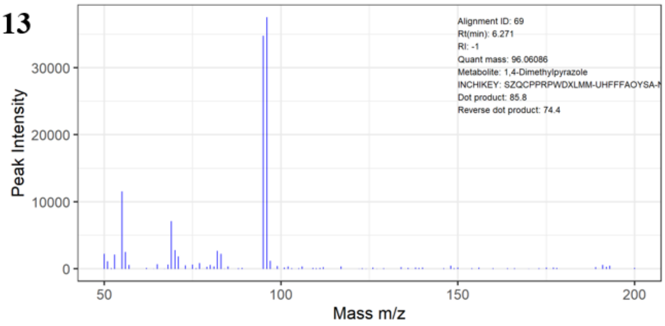

14

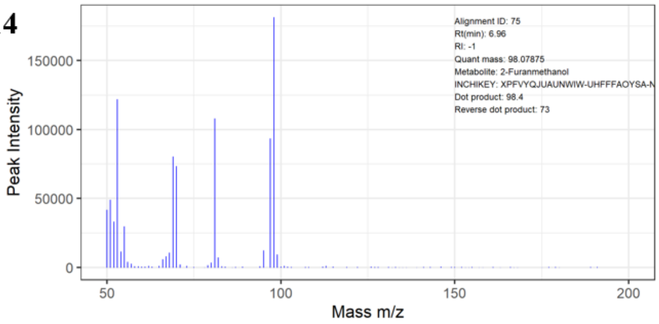

15

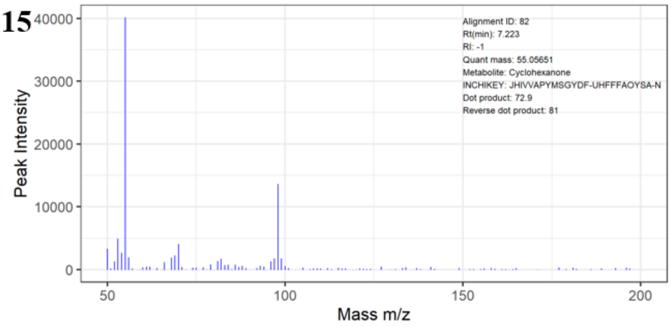

16

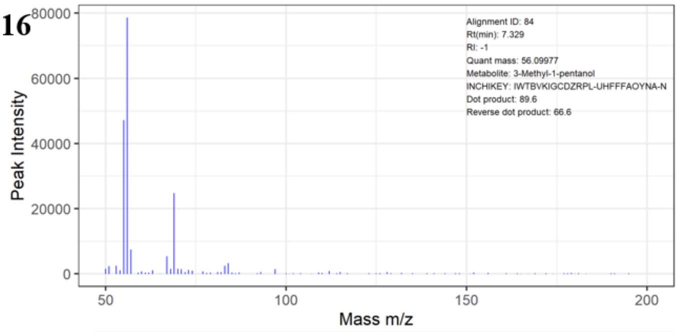

17

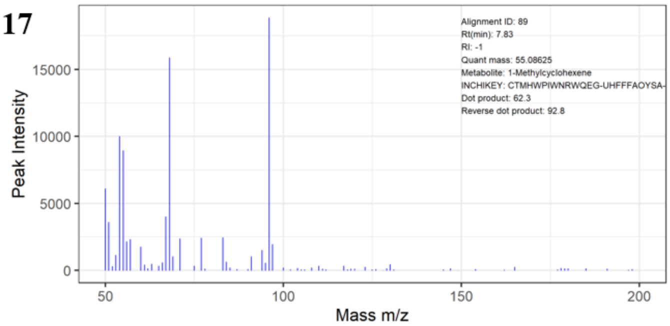

18

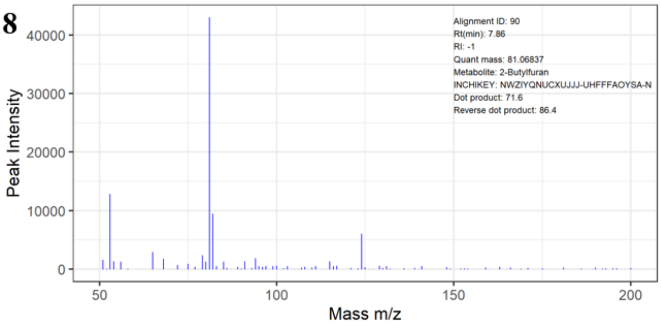

19

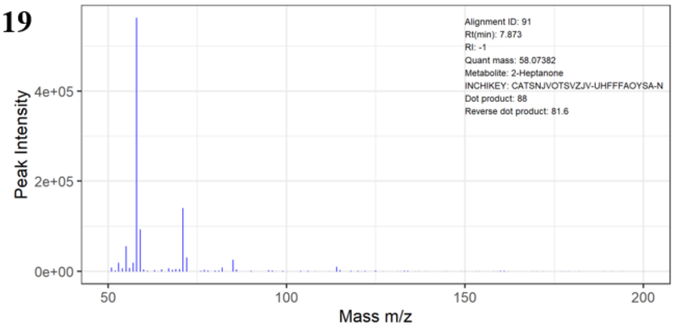

20

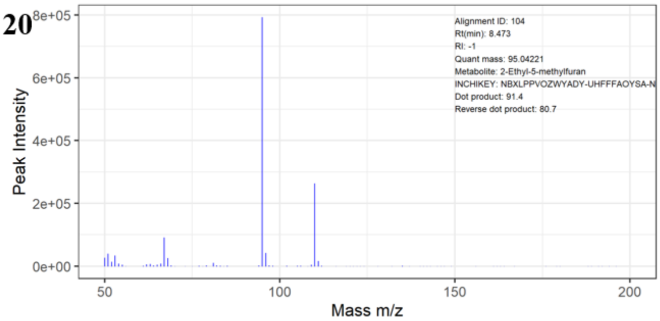

21

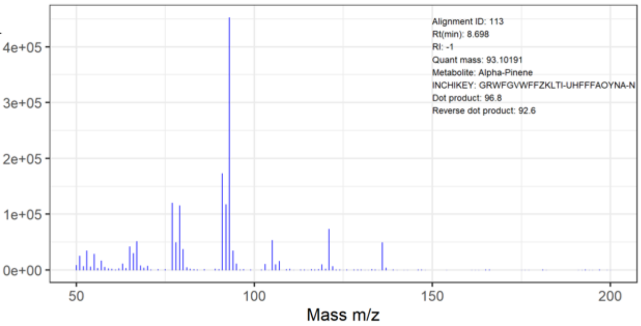

22

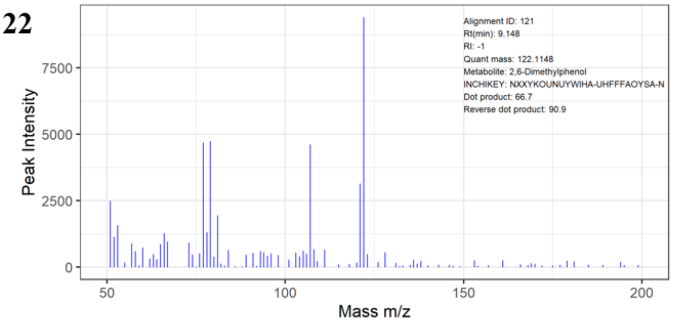

23

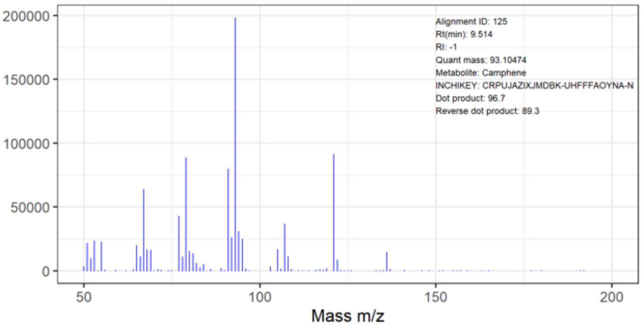

24

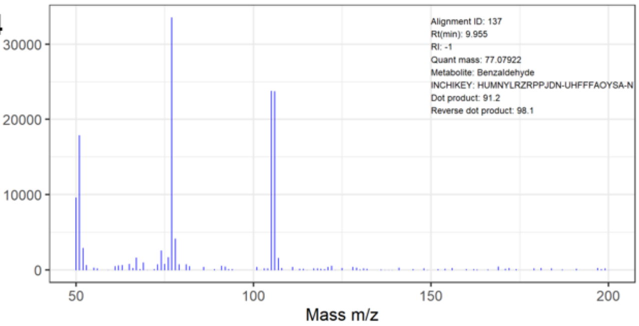

25

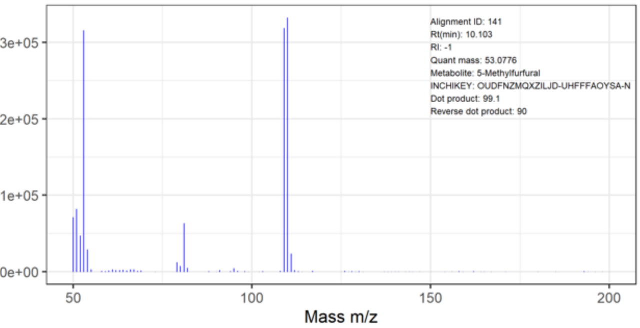

26

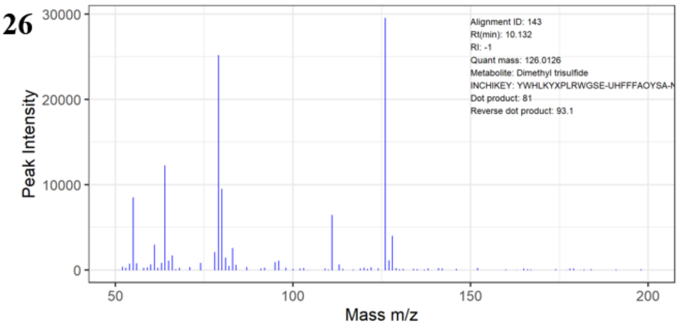

27

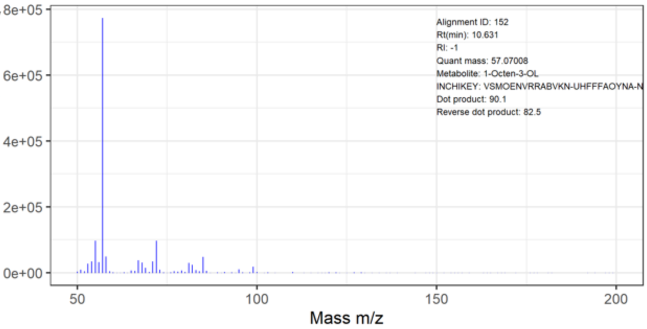

28

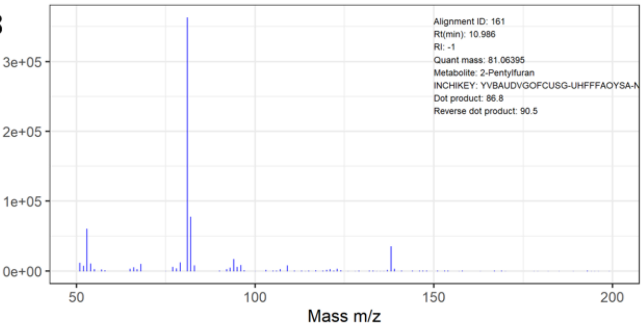

29

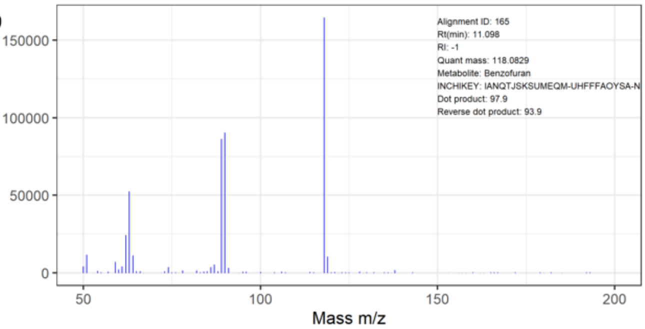

30

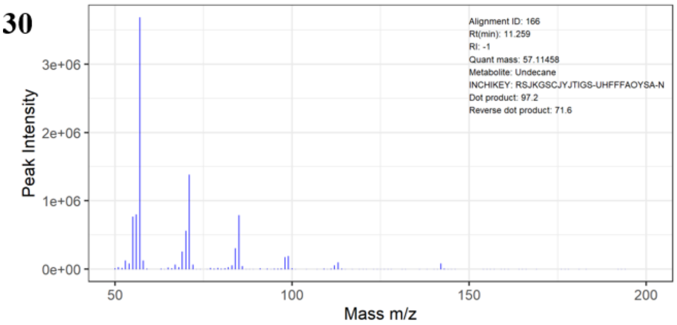

31

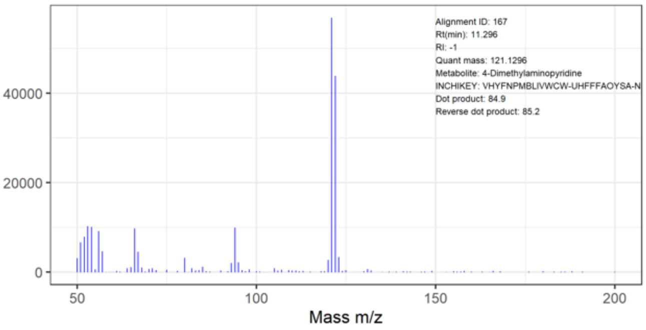

32

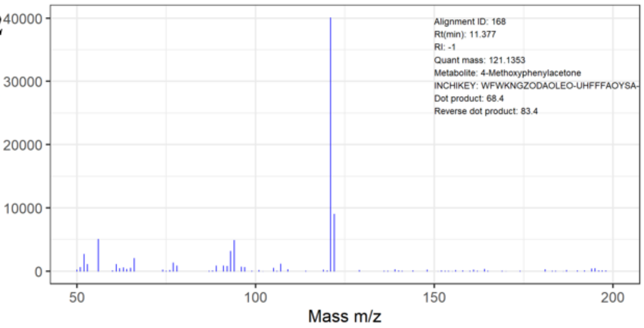

33

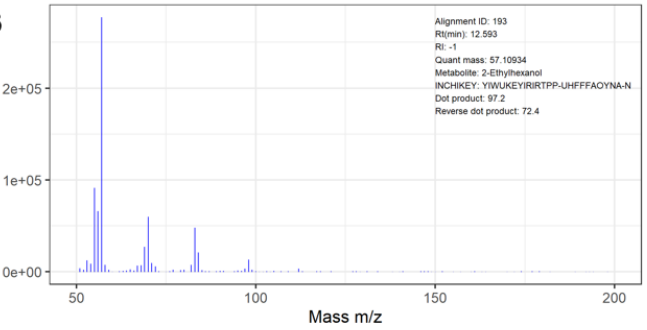

34

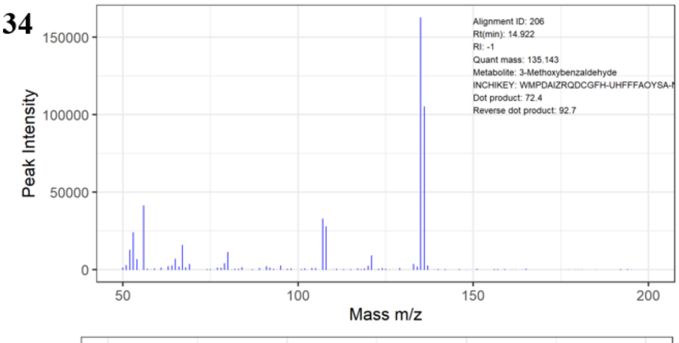

35

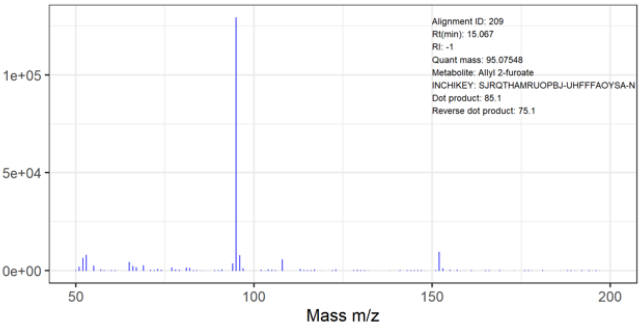

36

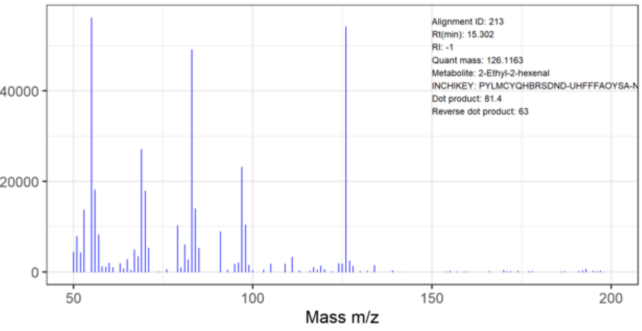

37

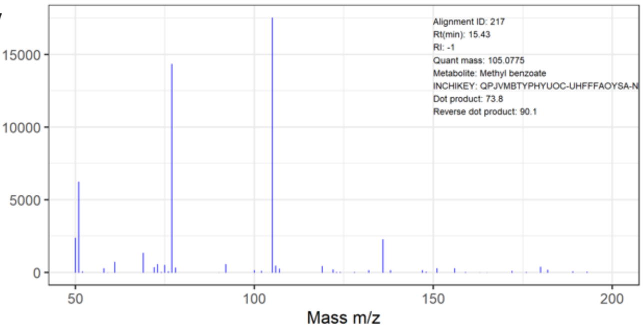

38

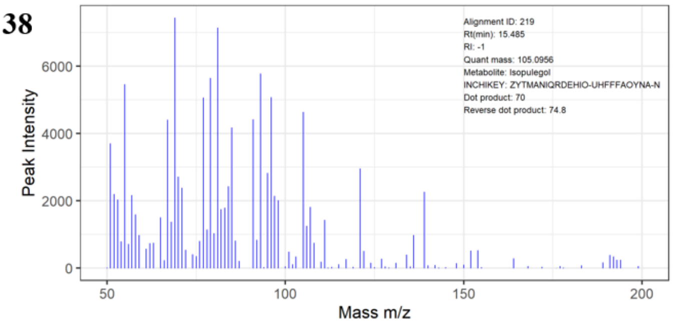

39

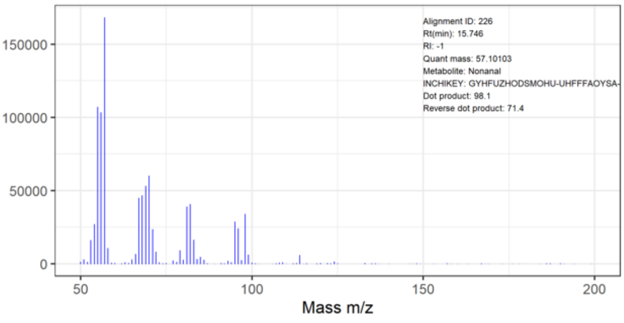

40

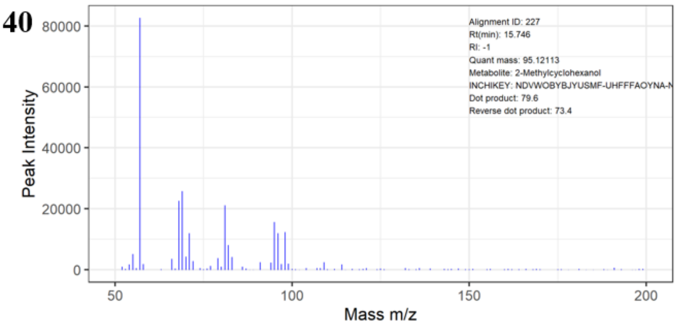

41

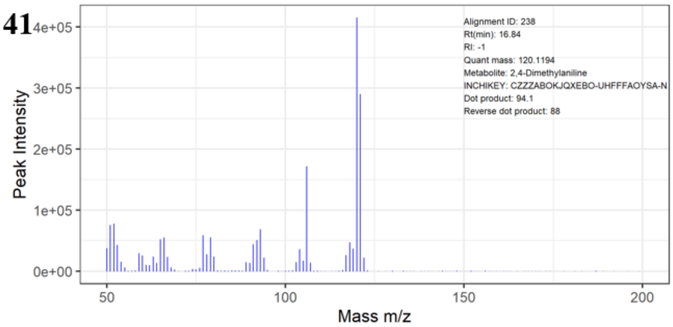

42

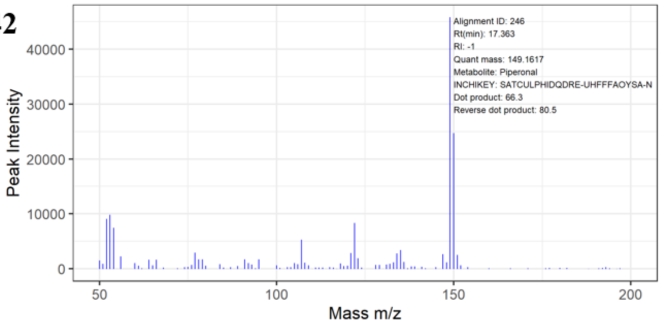

43

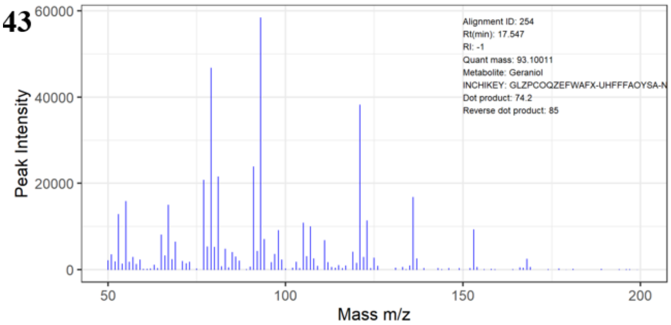

44

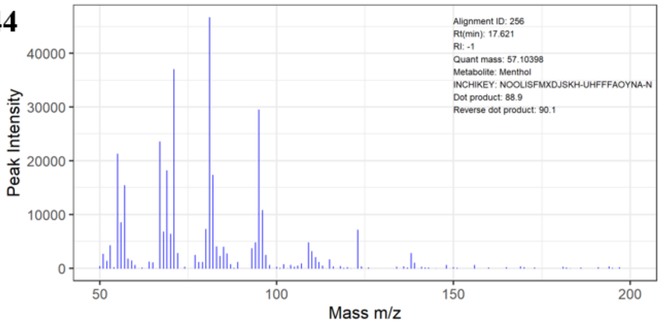

45

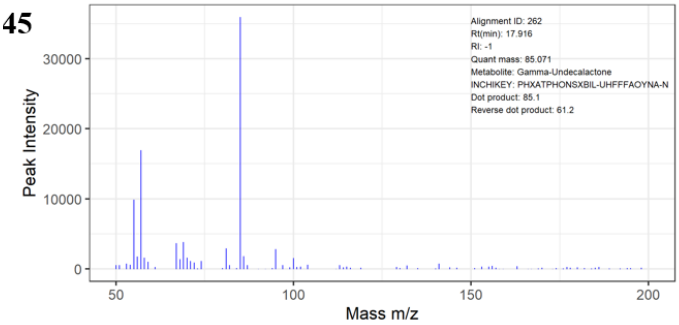

46

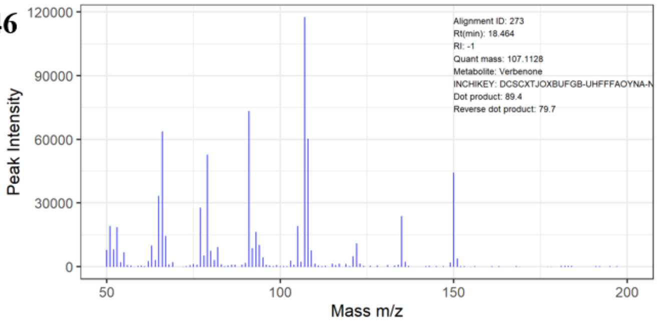

47

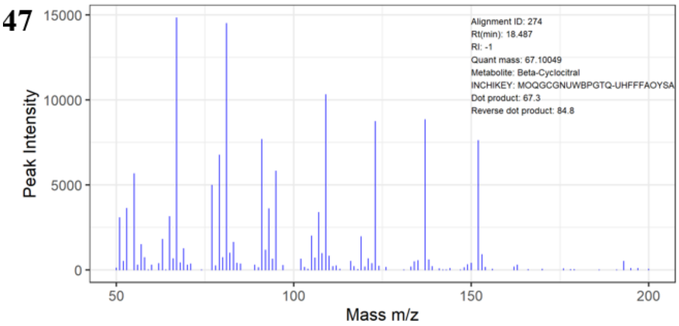

48

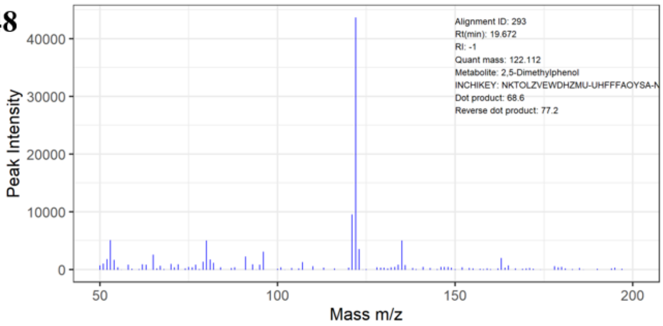

49

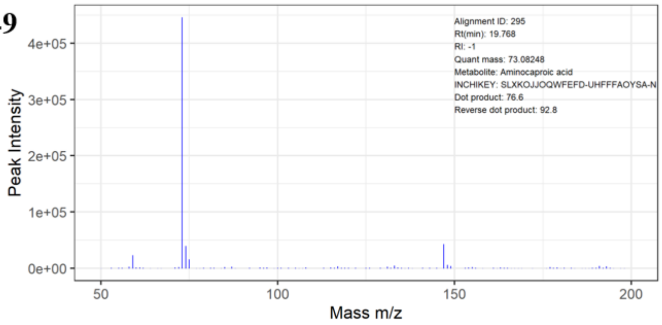

50

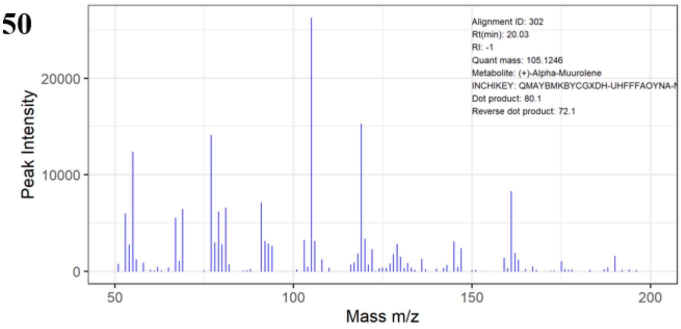

51

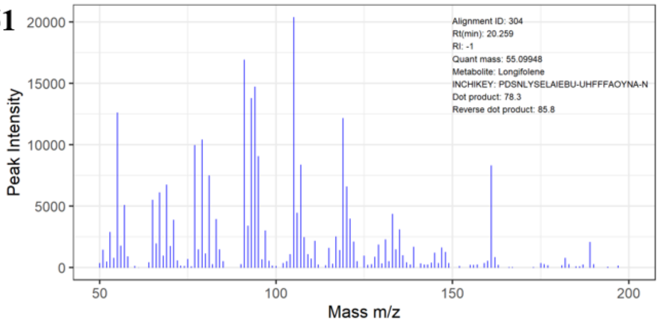

52

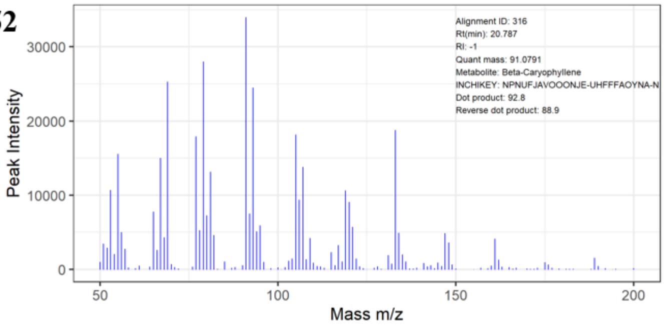

53

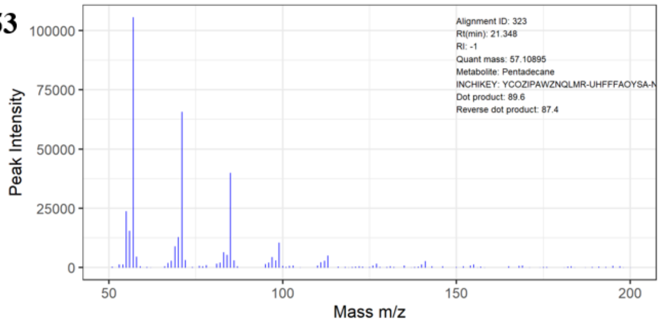

54

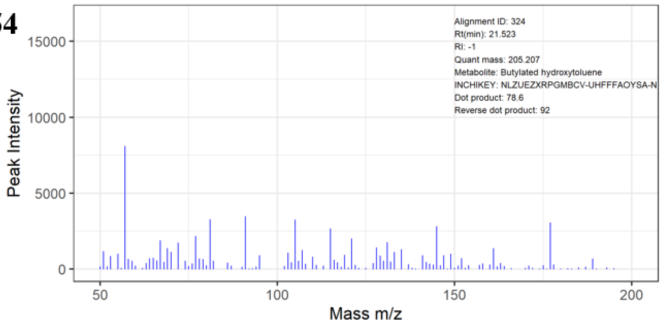

Figure S4. Mass spectra of 54 identified compounds.

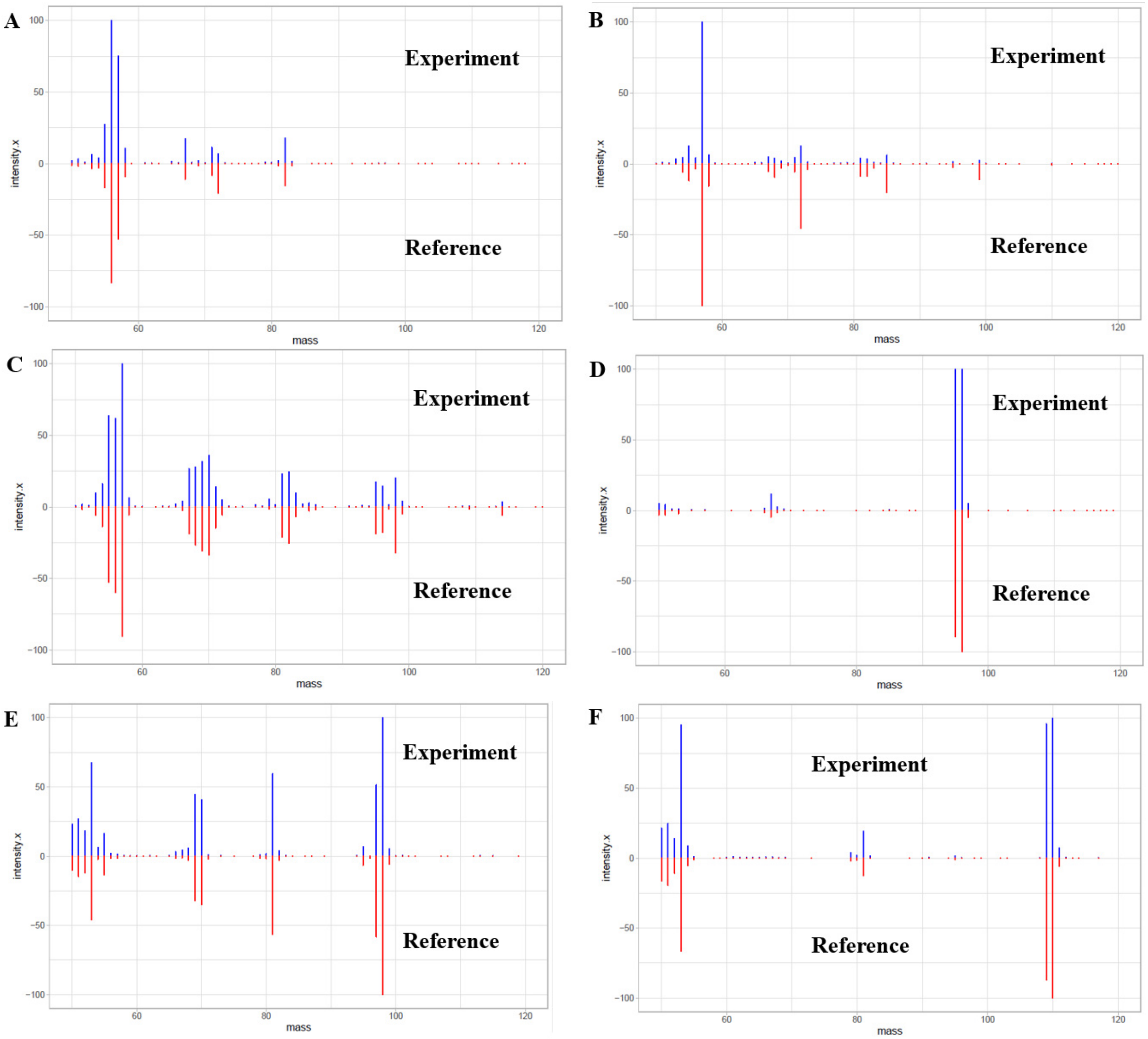

Figure S5. Identification of 6 key flavor compounds. (A) Hexanal. (B) 1-Octen-3-OL. (C) Nonanal. (D) Furfural. (E) 2-Furanmethanol. (F) 5-Methylfurfural.
